# Do oleic acid–enriched oil formulations improve health outcomes in metabolic disorders? A GRADE-assessed meta-analysis

**DOI:** 10.3389/fnut.2026.1766489

**Published:** 2026-06-24

**Authors:** Junyan Hua, Mengsi Hu, Yingjian Wang, Zhiwei Liu, Yanzhong Wang

**Affiliations:** 1 Department of Biochemical Engineering, University College London, London, United Kingdom; 2 Department of Blood Transfusion, Sir Run Run Shaw Hospital, Zhejiang University School of Medicine, Hangzhou, Zhejiang, China; 3 Department of Clinical Laboratory, Sir Run Run Shaw Hospital, Zhejiang University School of Medicine, Hangzhou, Zhejiang, China; 4 School of Medicine, Shaoxing University, Shaoxing, Zhejiang, China

**Keywords:** blood pressure, glycemic control, lipid profile, obesity measures, oleic acid

## Abstract

**Background:**

Oleic acid, a monounsaturated fatty acid (MUFA), exhibits beneficial properties in healthy subjects. However, it remains unclear whether these favorable effects extend to metabolically unhealthy individuals. Therefore, this study aimed to investigate the effect of an oleic acid-enriched oil formula on metabolic health outcomes.

**Methods:**

Our comprehensive systematic search was conducted in the following databases: PubMed, Scopus, Web of Science, and Google Scholar based on PRISMA guideline. The random-effect model was used to pool standardized mean differences (SMD).

**Results:**

Fifteen eligible studies met our inclusion criteria and were included in our systematic review and meta-analysis. Data analysis indicated that oleic acid consumption is not associated with improvement of lipid markers including total cholesterol (TC), low density lipoprotein cholesterol (LDL-c), high density lipoprotein cholesterol (HDL-c), and triglyceride (TG). In addition, it has been shown that subgroup analysis based on the BMI of participants, intervention duration, and oleic acid content could not modify the response to oleic acid intervention in term of lipid profile. Moreover, combined effect of studies evaluating the effect of the oleic acid on the glycemic control, obesity measures, and blood pressure illustrated that oleic acid is not potent enough to affect them in unhealthy individuals.

**Conclusion:**

The current meta-analysis indicated that oleic acid intervention exerts population specific responses; despite its beneficial properties, oleic acid does not improve health outcomes in metabolically unhealthy individuals.

## 1 Introduction

Metabolic disorders include wide range of disorders such as insulin resistance, low-grade inflammation which are associated with increased risk of noncommunicable diseases (NCDs) including cardiovascular diseases, dyslipidemia, type 2 diabetes, and hypertension (1, 2). Previous studies indicated that sedentary lifestyle and calorie dense diets are highly associated with elevated risk of NCDs (3, 4). This issue highlights the importance of dietary modifications across NCDs risk (5). It has been shown that early nutritional intervention may decrease the prevalence and progression of metabolic disorders. In this context, previous studies underscore the beneficial properties of the dietary fatty acids specifically oleic acid (6).

Oleic acid is a monounsaturated fatty acid (MUFA) and is known as omega-9 (7). It is comprised of 18 carbon atoms with a single cis-double bond (7). It has been shown that oleic acid is rich in olive oil, avocado, nuts (8). Evidence suggest that oleic acid may have cardioprotective properties through affecting lipid profile (9). Some studies have shown that it is able to reduce low-density lipoprotein cholesterol (LDL-c) level (9, 10). Moreover, one randomized trial has attributed antihypertensive effects to this fatty acid too (11). Improved endothelial and blood pressure may exert antihypertensive effects (11). Furthermore, several studies have reported that oleic acid may have critical role in the management of glycemic control (12, 13). This action may be possible through insulin sensitivity and anti-inflammatory properties (13).

However, findings across clinical trials remain inconsistent. Therefore, a systematic review and meta-analysis is warranted to evaluate the efficacy of an oleic acid-enriched oil formulation on metabolic health in individuals with metabolic disturbances.

## 2 Methods and materials

### 2.1 Search strategy

The present meta-analysis was conducted in adherence to Preferred Reporting Items for Systematic Reviews and Meta-Analyses (PRISMA) guidelines (14). The study protocol was registered in the PROSPERO (CRD420261388423).

### 2.2 Screening process and inclusion criteria

The electronic databases including PubMed, Scopus, and Web of Sciences were used for systematic search. In addition, the references of related studies were checked for inclusion of any relevant study. No language restriction was conducted. The search strategy was completed using MESH terms and keywords. The screening process was done by two researchers independently and any disagreement was resolved by third investigator.

The inclusion criteria were defined based on PICO statement; (1) Population (P); Adults with metabolic disorders; (2) Intervention (I); Oleic acid-enriched oil formulation, Comparison (C); control group, Outcome (O); Lipid profile (TC, LDL-c, HSL-c, TG), Glycemic markers (FBS, fasting insulin), anthropometric measurements (weight, BMI), and blood pressure (SBP, DBP). Healthy individuals, individuals with regular exercise, observational or quasi-experimental designs, and studies that did not provide sufficient data were excluded from the analysis.

### 2.3 Data extraction

Data extraction process was completed based on predefined inclusion criteria. Basic characteristics of all included studies were extracted such as: first author’s name, publication year, country, health condition, study design, sample size, mean age, mean BMI, intervention type, and follow-up duration.

### 2.4 Quality assessment

The quality assessment of all included studies was performed using Cochrane Collaboration’s risk of bias tool for randomized trials (RoB 2) by two independent researchers (15). The risk of bias was rated as lower, higher risk of bias, or some concerns. In addition, the GRADE-assessment tool was used to evaluate the quality of evidence too.

### 2.5 Statistical methods

The EndNote software was used to manage references. Also, the STATA software (version 18) was used to run statistical analysis. The association between oleic acid consumption and health outcomes was investigated among adults except healthy subjects and individuals with regular exercise program. The Cochrane *Q* test and the *I*^2^ statistic were used to show heterogeneity across studies.

## 3 Results

### 3.1 Study selection process

**Figure 1:**
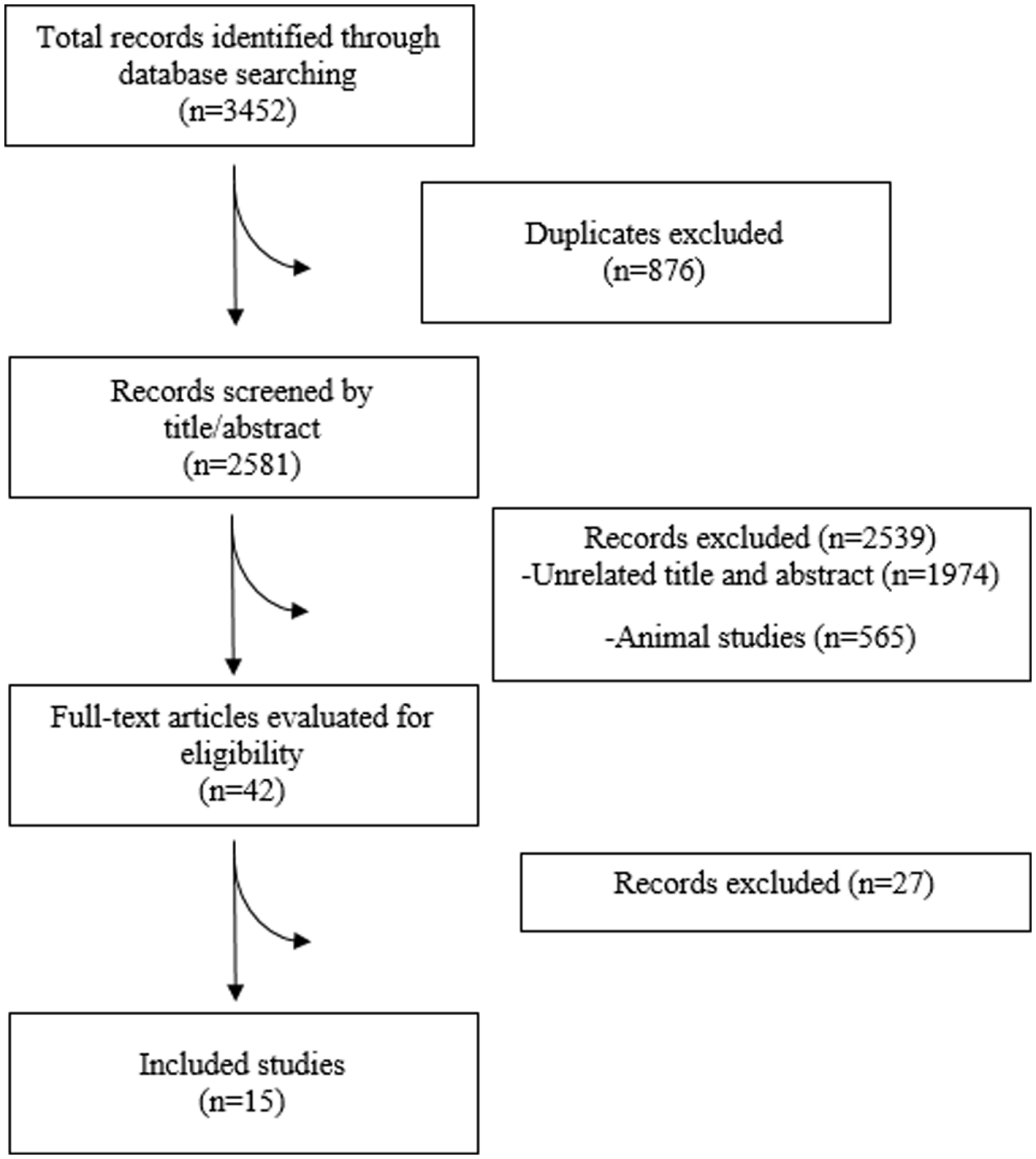
The flow diagram of the PRISMA study selection process.

The database searching process ended in 3,452 records. In addition, 5 records were retrieved from manual searching of the reference list of relevant studies. After removing 876 duplicates, 2,581 publications were screened using title and abstract. Then, 42 eligible studies were evaluated based on full-texts and 27 studies were excluded by reason such as: irrelevant studies, non-randomized trials, and studies which had not provided sufficient data. Finally, 15 eligible studies were included in our meta-analysis based on prespecified inclusion criteria. The flow diagram of study selection process is provided in the Figure 1.

### 3.2 Basic characteristics of included studies

The included studies were published between 1990 and 2023. Adults with different health disorders were included in the current study. Most of the included studies (12 trials) had parallel study design and three were crossover. The sample size was varied between 18 and 260 participants. In addition, study duration included wide range from 3 week to 24 weeks. Furthermore, this study included participants with various metabolic disorders such as: individuals with obesity, polycystic ovarian syndrome, hypercholesterolemia, cardiovascular disease risk (see Table 1).

**Table 1:** Basic characteristics of all included studies.

First author, publication year	Location	Health condition	Study design	Sample size (Int/Con)	Mean age (year)	BMI (kg/m^2^)	Intervention	Control	Follow up (week)
González-Rámila et al. (2023) (35)	Spain	Healthy subjects and at-risk	Crossover	64, 64	44	24	76.5% of high-oleic acid sunflower oil	71% of oleic acid of olive pomace oil	4
González-Rámila et al. (2022) (34)	Spain	Healthy subjects and at-risk	Crossover	68, 68	30	23	74.32% of oleic acid of olive pomace oil	29.76% of oleic acid of sunflower oil	4
Caldas et al. (2020) (30)	Brazil	Overweight men	Parallel	21, 21	27.6	27.5	High-oleic peanut [oleic acid (C18:1n9) content of 81.5%]	Conventional peanut [oleic acid (C18:1n9) content of 51%]	4
Bowen et al. (2019) (29)	Canada	Adults with central adiposity	Crossover	119, 119	44	31.5	70% of high-oleic acid canola oil	60% of oleic acid of conventional canola oil	6
Liu et al. (2018) (37)	Canada	Population with or at risk for metabolic syndrome	Crossover	101, 101	49.5	29.5	72% of high-oleic acid canola oil	62% of oleic acid of conventional canola oil	4
Harris et al. (2017)	USA	Postmenopausal women	Crossover	20, 20	58.8	26.5	80% of high-oleic acid sunflower oil	3% of oleic acid of virgin coconut oil	4
Pu et al. (2016) (38)	Canada	Participants with cardiovascular disease risk	Crossover	84, 84	45.6	29.5	72% of high-oleic acid canola oil	60% of oleic acid of conventional canola oil	4
Jones et al. (2015) (9)	Multicenter	Individuals with abdominal obesity	Crossover	50, 50	45.8	30.5	71.5% of high oleic canola oil	17.6% of oleic acid of blend of corn/safflower oil	4
Jones et al. (2014) (36)	Multicenter	Adult with at least one of the cardiovascular risk factors	Crossover	130, 130	46.4	29.5	71.5% of high oleic canola oil	58.6% of oleic acid of regular canola oil	4
Alves et al. (2014) (27)	Brazil	Overweight/obese	Parallel	21, 22	27.4	29.5	81.5% of high oleic peanut	51% of oleic acid of regular peanuts	4
Gillingham et al. (2012) (33)	Canada	Hypercholesterolemic subjects	Crossover	34, 34	35.2	28.12	High-oleic acid canola oil (HOCO) [oleic acid (C18:1n9) content of 70%]	Typical western diet (WD)	4
Gillingham et al. (2011) (32)	Canada	Hypercholesterolemia	Crossover	36, 36	47.49	28.5	73.7% of high-oleic acid canola oil	46.5% of oleic acid of typical western diet	4
Zambon et al. (1999) (40)	Italy	Mildly obese women	Parallel	9, 11	30	31	75.5% of olive-oil-enriched hypocaloric diet	10–20% of oleic acid of carbohydrate enriched hypocaloric diet	24
Cater et al. (1997) (31)	USA	Middle-aged men with mild hypercholesterolemia	Crossover	9, 9	66	27	87% of high-oleic acid sunflower oil	35% of oleic acid of palmolein	3
Wardlaw et al. (1990) (39)	USA	Healthy subjects or at-risk	Crossover	86, 86	NR	NR	85% of high-oleic acid sunflower oil	20–30% of oleic acid of corn oil	5

### 3.3 Oleic acid–enriched oil and lipid profile

#### 3.3.1 TC

**Figure 2:**
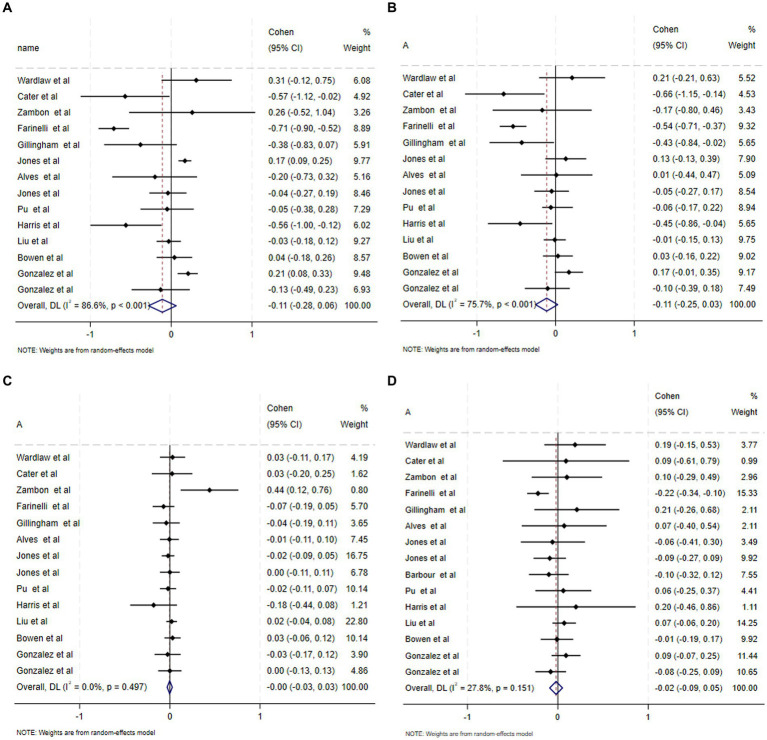
**(A)** The forest plot of the oleic acid-enriched formula on TC levels. **(B)** The forest plot of the oleic acid-enriched formula on LDL-c levels. **(C)** The forest plot of the oleic acid-enriched formula on HDL-c levels. **(D)** The forest plot of the oleic acid-enriched formula on TG levels.

Fourteen studies have evaluated the effect of oleic acid-enriched oil formula on the TC level and there was no significant association accordingly (WMD: −0.11; 95% CI: −0.28 to 0.06; *p* = 0.212, *I*^2^ = 86.6%; *p* < 0.001) (Figure 2A). Subgroup analysis based on the BMI, duration, and oleic acid content from total fatty acid did not show any significant changes following oleic acid consumption (Table 2). Sensitivity analysis demonstrated that excluding Zambon et al. (40) studies could affect the pooled estimate (Supplementary Figure 1). In an additional sensitivity analysis excluding studies with <10% absolute difference in oleic acid content between intervention and control diets, the pooled effect for TC remained non-significant.

**Table 2:** Subgroup analysis of lipid profile markers.

Outcomes	No. of comparisons	Effect size (95% CI)	*p*-value	*I*^2^-heterogeneity (%)	*p*-value
TC					
*BMI*					
≤30	3	−0.21 (−0.86, 0.44)	0.530	96.8	<0.001
>30	10	−0.08 (−0.23, 0.07)	0.300	67.1	0.001
NR	1	0.31 (−0.13, 0.75)	0.162	0.0	<0.001
*Duration (week)*					
≤4	10	−0.06 (−0.20, 0.08)	0.404	72.3	<0.001
>4	4	−0.07 (−0.61, 0.48)	0.813	91.8	<0.001
*Oleic intake from total fatty acid*					
<80%	10	0.00 (−0.12, 0.12)	0.955	65.4	0.002
≥80%	4	−0.31 (−0.81, 0.20)	0.238	84.4	<0.001
LDL-C					
*BMI*					
≤30	3	−0.16 (−0.63, 0.31)	0.510	93.8	<0.001
>30	10	−0.09 (−0.20, 0.03)	0.145	42.3	0.075
NR	1	0.21 (−0.21, 0.63)	0.327	0.0	<0.001
*Duration (week)*					
≤4	10	−0.07 (−0.20, 0.05)	0.245	55.7	0.016
>4	4	−0.13 (−0.53, 0.27)	0.513	87.6	<0.001
*Oleic intake from total fatty acid*					
<80%	10	−0.03 (−0.13, 0.07)	0.571	38.3	0.103
≥80%	4	−0.26 (−0.66, 0.15)	0.217	79.8	0.002
HDL-C					
*BMI*					
≤30	3	−0.03 (−0.11, 0.04)	0.372	0.0	0.732
>30	10	0.00 (−0.03, 0.04)	0.902	16.4	0.292
NR	1	0.03 (−0.11, 0.17)	0.674	0.0	<0.001
*Duration (week)*					
≤4	10	−0.01 (−0.04, 0.03)	0.678	0.0	0.963
>4	4	0.04 (−0.08, 0.17)	0.474	66.1	0.031
*Oleic intake from total fatty acid*					
<80%	10	0.00 (−0.03, 0.04)	0.817	13.7	0.137
≥80%	4	−0.02 (−0.07, 0.03)	0.465	0.0	0.727
TG					
*BMI*					
≤30	3	−0.08 (−0.26, 0.11)	0.425	76.5	0.010
>30	11	0.01 (−0.07, 0.08)	0.803	0.0	0.917
NR	1	0.19 (−0.15, 0.53)	0.273	0.0	<0.001
*Duration (week)*					
≤4	10	0.02 (−0.05, 0.09)	0.559	0.0	0.806
>4	5	−0.06 (−0.20, 0.08)	0.416	53.1	0.076
*Oleic intake from total fatty acid*					
<80%	11	0.01 (−0.05, 0.08)	0.730	0.0	0.778
≥80%	4	−0.06 (−0.28, 0.16)	0.571	48.5	0.120

#### 3.3.2 LDL-c

The pooled estimate of fourteen studies indicated that oleic acid-enriched oil formula could not affect LDL-c level significantly (WMD: −0.11; 95% CI: −0.25 to 0.03; *p* = 0.116, *I*^2^ = 75.7%; *p* < 0.001) (Figure 2B). However, subgroup analysis based on critical subgroups such as BMI, treatment duration, and amount of oleic acid from total fatty acid did not show significant alteration following treatment (Table 2). In addition, sensitivity analysis using leave-one-out approach showed that no study is able to affect the pooled analysis (Supplementary Figure 2). After excluding studies with <10% absolute difference in oleic acid content, the result for LDL-c remained non-significant.

#### 3.3.3 HDL-c

The combined effect of fourteen publications evaluating the effect of oleic acid-enriched oil formula demonstrated that it is not potent enough to affect HDL-c level among adults with health disorders (WMD: −0.00; 95% CI: −0.03 to 0.03; *p* = 0.914, *I*^2^ = 0.0%; *p* = 0.497) (Figure 2C). Also, subgroup analysis showed that the intervention failed to exert significant effect in each subgroup based on BMI, intervention duration, and oleic acid content of the formula (Table 2). Interestingly, sensitivity analysis illustrated that no study is able to alter the final result which suggests that robustness of the findings (Supplementary Figure 3). The sensitivity analysis excluding low-contrast studies (<10% oleic acid difference) also showed no significant change for HDL-c.

#### 3.3.4 TG

Fifteen publications had evaluated the efficacy of the oleic acid-enriched oil formula on the TG level and no significant changes was observed subsequently (WMD: −0.02; 95% CI: −0.09 to 0.05; *p* = 0.586, *I*^2^ = 27.8%; *p* = 0.151) (Figure 2D). No significant results were shown based on subgroup analysis too (Table 2). Moreover, sensitivity analysis indicated that excluding single study is not able to change the overall result and this result highlights the robustness of the findings (Supplementary Figure 4). Furthermore, excluding studies with <10% absolute difference in oleic acid content did not alter the null finding for TG.

### 3.4 Oleic acid–enriched oil and glycemic control

#### 3.4.1 FBS

**Figure 3:**
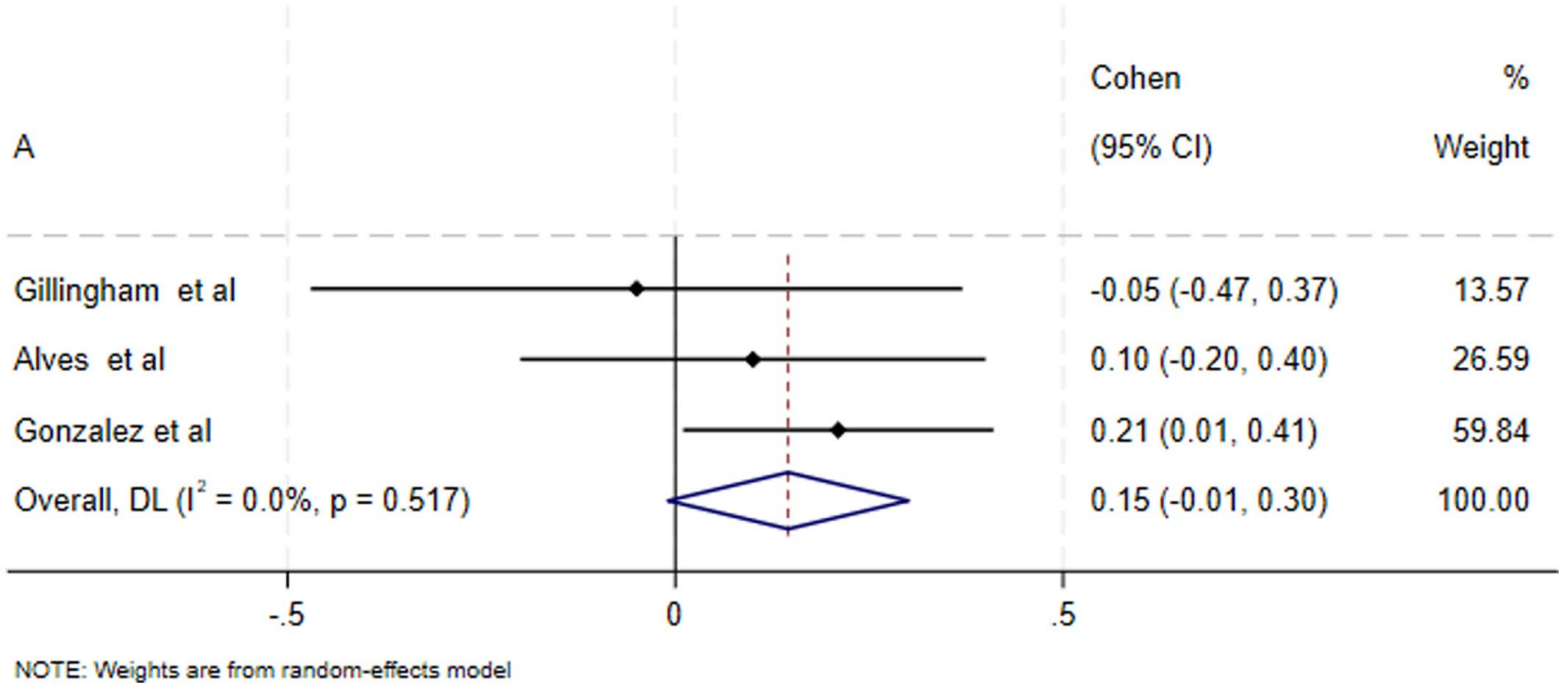
The forest plot of the oleic acid-enriched formula on FBS levels.

Three studies containing patients with metabolic disorders have evaluated the effect of the oleic acid on the FBS level. The pooled effect of these studies has revealed that oleic acid-enriched oil formulation could not affect FBS level significantly (WMD: 0.15; 95% CI: −0.01 to 0.30; *p* = 0.065, *I*^2^ = 0.0%; *p* = 0.517) (Figure 3). While, sensitivity analysis showed that excluding Gillingham et al’ study would affect the overall result and change it to significant mode (Supplementary Figure 5).

#### 3.4.2 Fasting insulin

Our systematic review included two studies which have reported the effect of oleic acid-enriched formula on the fasting insulin level. Alves et al.’s (27) study concluded that this formulation would reduce fasting insulin level but it did not reach statistical significancy.

### 3.5 Oleic acid–enriched oil and anthropometric measurements

#### 3.5.1 Weight

**Figure 4:**
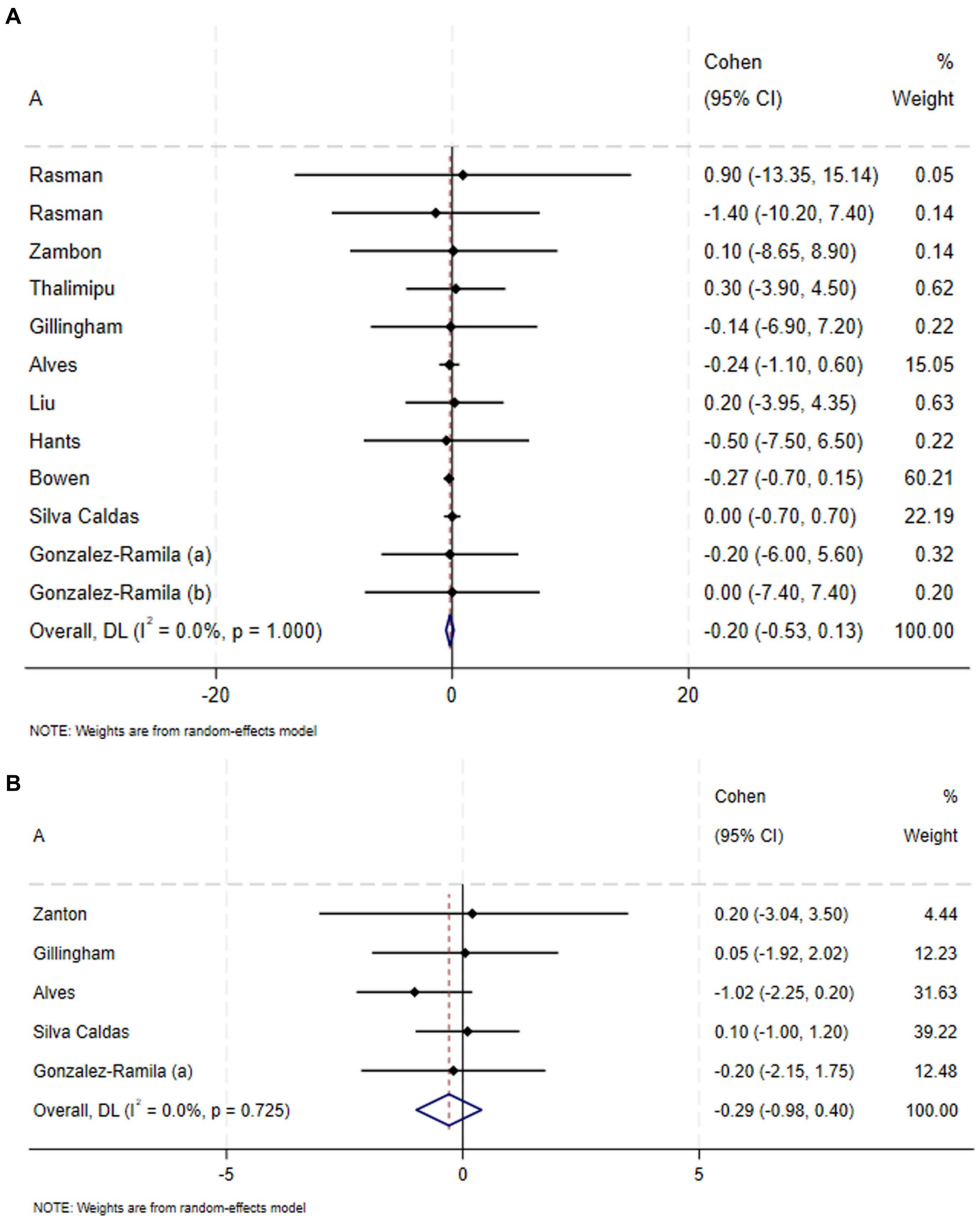
**(A)** The forest plot of the oleic acid-enriched formula on weight levels. **(B)** The forest plot of the oleic acid-enriched formula on BMI levels.

The pooled estimate of twelve studies evaluating the effect of oleic acid-enriched oil on weight measurement indicated that there is no association between oleic acid content of oil formula and weight value (SMD: −0.20; 95% CI: −0.53 to 0.13; *p* = 0.237, *I*^2^ = 0.0%; *p* = 1.00) (Figure 4A). In addition, sensitivity analysis indicates that exclusion of any single study could not change the overall result (Supplementary Figure 6).

#### 3.5.2 BMI

The combined effect of five studies involving 301 participants showed that oleic acid consumption is not associated with BMI level significantly (SMD: −0.29; 95% CI: −0.98 to 0.40; *p* = 0.404, *I*^2^ = 0.0%; *p* = 0.725) (Figure 4B). Sensitivity analysis using leave-one-out approach revealed that no study is able to affect the pooled estimate of BMI variable (Supplementary Figure 7).

### 3.6 Oleic acid–enriched oil and blood pressure

#### 3.6.1 SBP

**Figure 5:**
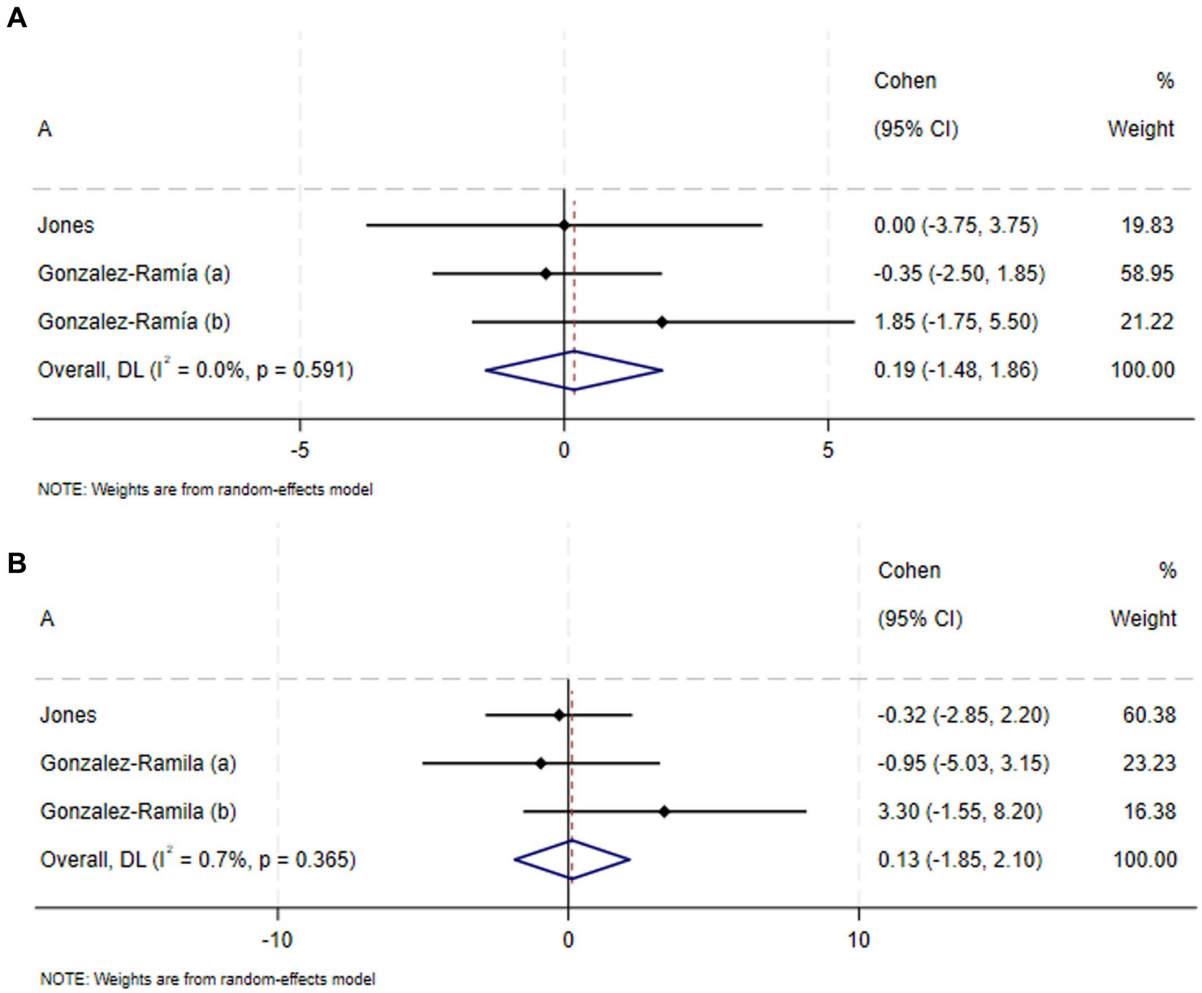
**(A)** The forest plot of the oleic acid-enriched formula on SBP levels. **(B)** The forest plot of the oleic acid-enriched formula on DBP levels.

Three studies encompassing 396 participants (intervention group = 262, control group = 262) evaluated the effect of the oleic acid-enriched formula on the SBP level among adults with health disorders and no significant effect was shown accordingly (SMD: 0.19; 95% CI: −1.48 to 1.86; *p* = 0.827, *I*^2^ = 0.0%; *p* = 0.591) (Figure 5A). Moreover, sensitivity analysis confirmed that exclusion of any single study is not able to affect the pooled estimate on SBP too (Supplementary Figure 8).

#### 3.6.2 DBP

Three studies involving 396 individuals evaluated the effect of the oleic acid-enriched formula on the DBP level among adults with health disorders and there was no significant effect on DBP level (SMD: 0.13; 95% CI: −1.85 to 2.10; *p* = 0.900, *I*^2^ = 0.7%; *p* = 0.365) (Figure 5B). In addition, sensitivity analysis approved this finding that the final effect of the oleic acid on the DBP may not be affected by excluding any single study (Supplementary Figure 9).

### 3.7 Risk of bias assessment and quality of evidence

The quality assessment of the included studies is provided in the Table 3. Most of the included studies were rated as lower risk of bias for randomization process, selection of the reported result, and missing outcome data domains. The deviation from intended intervention obtained higher risk of bias or some concerns.

**Table 3:** Quality of included studies in the meta-analysis.

Study, year	Randomization process	Deviation from intended interventions	Selection of the reported result	Measurement of the outcome	Missing outcome data
González-Rámila et al. (2023) (35)	L	Some concern	L	Some concern	L
González-Rámila et al. (2022) (34)	L	H	L	Some concern	L
Caldas et al. (2020) (30)	Some concern	L	L	L	L
Bowen et al. (2019) (29)	L	H	L	Some concern	L
Liu et al. (2018) (37)	L	Some concern	L	Some concern	L
Harris et al. (2017)	L	Some concern	L	Some concern	L
Pu et al. (2016) (38)	L	H	L	H	L
Jones et al. (2015) (9)	L	H	Some concern	Some concern	L
Alves et al. (2014) (27)	L	H	L	Some concern	L
Jones et al. (2014) (36)	L	H	Some concern	Some concern	L
Gillingham et al. (2012) (33)	L	Some concern	L	Some concern	L
Gillingham et al. (2011) (32)	L	H	L	H	H
Zambon et al. (1999) (40)	L	Some concern	L	L	L
Cater et al. (1997) (31)	L	Some concern	Some concern	Some concern	L
Wardlaw et al. (1990) (39)	L	L	Some concern	L	Some concern

H, high risk of bias; L, low risk of bias.

Moreover, the quality of the evidence was evaluated using GRADE tool (Table 4) and most of the study outcomes (HDL-c, TG, weight, BMI, SBP, and DBP) obtained moderate score. While, TC, LDL-c, and FBS got low score for quality of evidence.

**Table 4:** Summary of GRADE assessment and quality of evidence.

Outcomes	No of studies	ES (95% CI)	Risk of bias	Inconsistency	Indirectness	Imprecision	Publication bias	Quality of evidence
TC	14	−0.11 (−0.28, 0.06)	Not serious	Serious	Not serious	Serious	Not serious	Low
LDL-c	14	−0.11 (−0.25, 0.03)	Not serious	Serious	Not serious	Serious	Not serious	Low
HDL-c	14	−0.00 (−0.03, 0.03)	Not serious	Not serious	Not serious	Serious	Not serious	Moderate
TG	15	−0.02 (−0.09, 0.05)	Not serious	Not serious	Not serious	Serious	Not serious	Moderate
FBS	4	0.01 (−0.22, 0.24)	Not serious	Serious	Not serious	Serious	Not serious	Low
Weight	12	−0.20 (−0.53, 0.13)	Not serious	Not serious	Not serious	Serious	Not serious	Moderate
BMI	5	−0.29 (−0.98, 0.40)	Not serious	Not serious	Not serious	Serious	Not serious	Moderate
SBP	3	0.19 (−1.48, 1.86)	Not serious	Not serious	Not serious	Serious	Not serious	Moderate
DBP	3	0.13 (−1.85, 2.10)	Not serious	Not serious	Not serious	Serious	Not serious	Moderate

### 3.8 Publication bias

The publication bias was assessed using Begg’s and egger’s tests. There was no evidence of publication bias in term of the lipid profile marker (TC, LDL-c, HDL-c, TG), fasting insulin, weight, BMI, SBP, and DBP (*p* > 0.05). Moreover, visual inspection of the funnel plots confirms this finding too (Supplementary Figures 10A–F). While, lower than ten studies were included for FBS variable, egger’s test was used to evaluate the publication bias and it was significant (*p* = 0.024) (Supplementary Figure 10G).

## 4 Discussion

The present comprehensive systematic review and meta-analysis summarized the effect of oleic acid-enriched oil formula on the metabolic health as well. It has been demonstrated that oleic acid-enriched oil formulation was not potent enough to exert significant beneficial effects on the lipid profile level among metabolically unhealthy individuals. Previous studies have shown that dietary patterns such as Mediterranean diet featuring oleic acid as a key component exert beneficial effects on lipid profile levels (14, 15). Additionally, canola oil‑based interventions have been associated with improvements in adiponectin concentrations, a marker of insulin sensitivity (28). This inconsistency suggests that the favorable effect of oleic acid on lipid profile may be masked in the patients with metabolic disorders. This population specific response highlights that the beneficial effects of the oleic acid may be blunted in metabolically unhealthy individuals. It seems that oleic acid is not potent enough to overcome the pathological changes in metabolic disturbances by insulin resistance, low-grade inflammation, and altered lipid metabolism. Previous studies suggest that the lipid lowering effect of the oleic acid may be possible through elevated activity of the acyl-CoA cholesterol acyltransferase (ACAT) in healthy individuals which contributes increased activity of the LDL receptors (16, 17). Insulin resistance seems to target this mechanism to be disrupted. Moreover, promoted inflammatory state in metabolic disorders interfere with protective properties of the oleic acid too (18, 19). However, subgroup analysis based on the BMI of participants, intervention duration, and oleic acid content from total fatty acid could show significant changes in term of the lipid profile (TC, LDL-c, HDL-c, TG). This finding underscores the inability of these subgroups to modify the response to oleic acid treatment, particularly in the setting of metabolic disorders.

In addition, this study evaluated the effect of oleic acid-enriched oil on the glycemic control (FBS, fasting insulin) too. In this context, it was unable to exerts its beneficial effect in unhealthy individuals across FBS and insulin level. This finding complements the null effect observed on lipid markers and may be driven by underlying metabolic disturbances. In contrast, healthy populations have demonstrated the efficacy of oleic acid in glycemic control, whereas it remains ineffective in unhealthy individuals (12, 20). The saturated fatty acid-induced inflammation is overwhelmed with oleic acid in healthy individuals (21, 22). However, metabolic disturbances, such as chronic and high-grade inflammatory states, diminish the beneficial effects of oleic acid (23).

It is worth noting that the beneficial effect of a dietary component is not assessed in isolation but rather depends on the clinical health condition of individuals undergoing treatment. Likewise, a holistic approach to evaluating the efficacy of dietary components reveals that metabolic disturbances also interfere with improvements in anthropometric variables.

Furthermore, this study demonstrated that oleic acid consumption is not associated with blood pressure improvement too. This finding aligns with previous null effects in term of lipid profile, glycemic state, and anthropometric measurements. This issue highlights that unhealth individuals are non-responsive to oleic acid intervention. It has been shown that oleic acid may exert its critical role through improved endothelial function, and elevated nitric oxide (NO) bioavailability (24, 25). Whereas, metabolically unhealthy individuals encounter with impaired NO signaling and endothelial dysfunction. Furthermore, evidence suggests that inflammatory signaling overcomes the protection afforded by oleic acid (23, 26).

This study has some limitations too. First, this study assessed the metabolically unhealthy individuals which include various health status and this issue increase the heterogeneity and limits the findings. Accordingly, the findings with higher heterogeneity should be interpreted with caution. Second, there was lack of individual patient dietary data. Third, number of trials in each subgroup based on health state, source of oleic acid was insufficient to conduct meaningful and reliable meta-regression and subgroup analyses.

## 5 Conclusion

The present systematic and meta-analysis summarized the effect of the oleic acid-enriched oil formula on the metabolic health and shared nuanced findings. It has been demonstrated that oleic acid consumption is not associated with an improvement of lipid profile, glycemic state, anthropometric indices and blood pressure in metabolically unhealthy individuals.

## Data Availability

The datasets presented in this study can be found in online repositories. The names of the repository/repositories and accession number(s) can be found in the article/Supplementary material.
