# Supplementary material for: Do oleic acid–enriched oil formulations improve health outcomes in metabolic disorders? A GRADE-assessed meta-analysis
Source: Front Nutr. 2026 Jun 24;13:1766489. doi: 10.3389/fnut.2026.1766489 (PMC13341285; doi:10.3389/fnut.2026.1766489)
Supplement: Supplementary file 1 [file Data_Sheet_1.PDF]

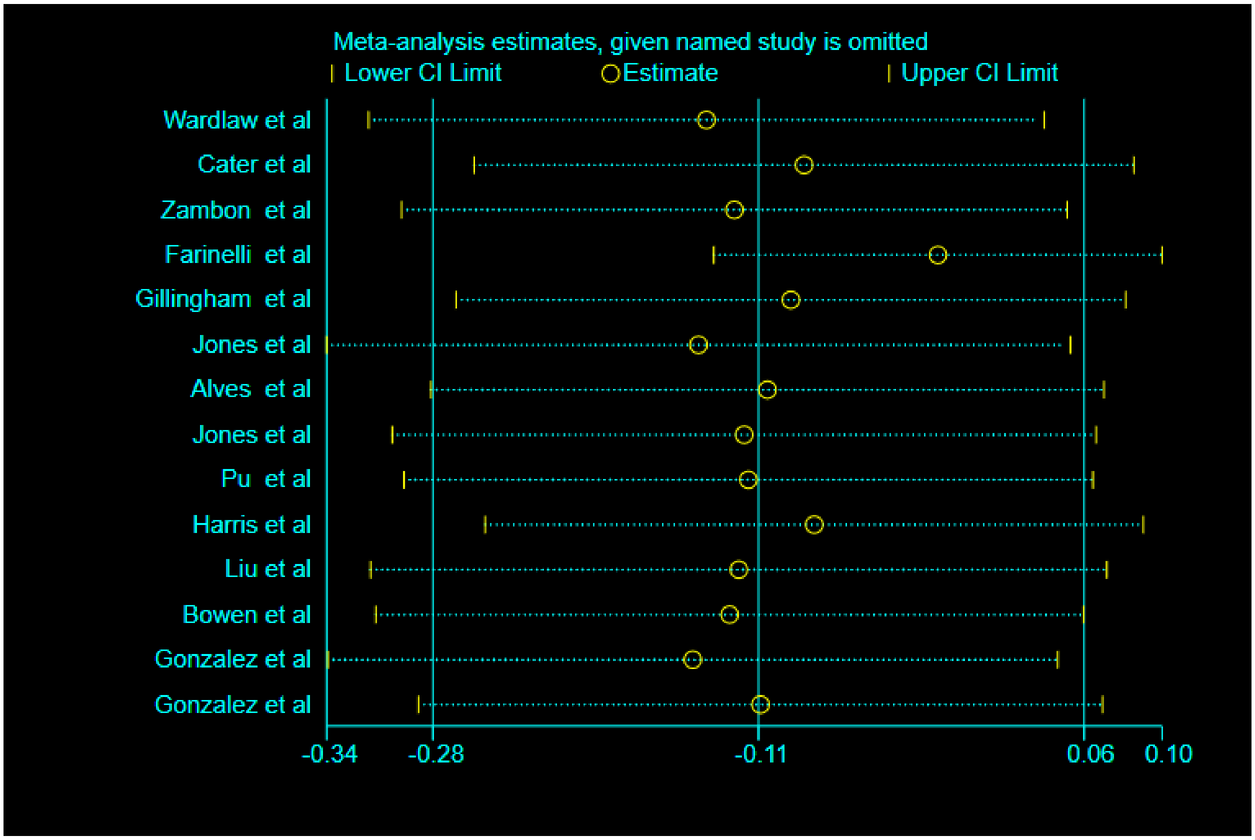

Supplementary Figure 1. TC

Supplementary Figure 2. LDL-C

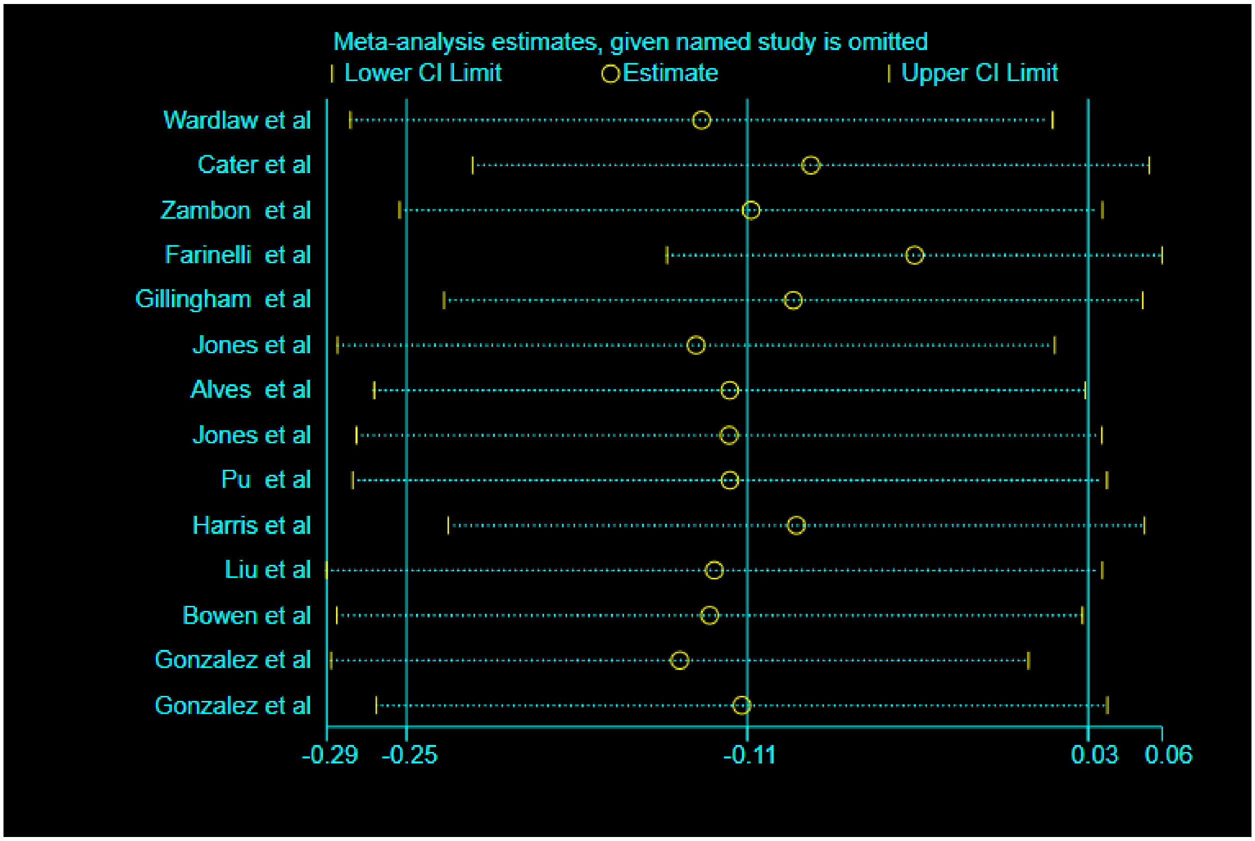

Supplementary Figure 3. HDL-C

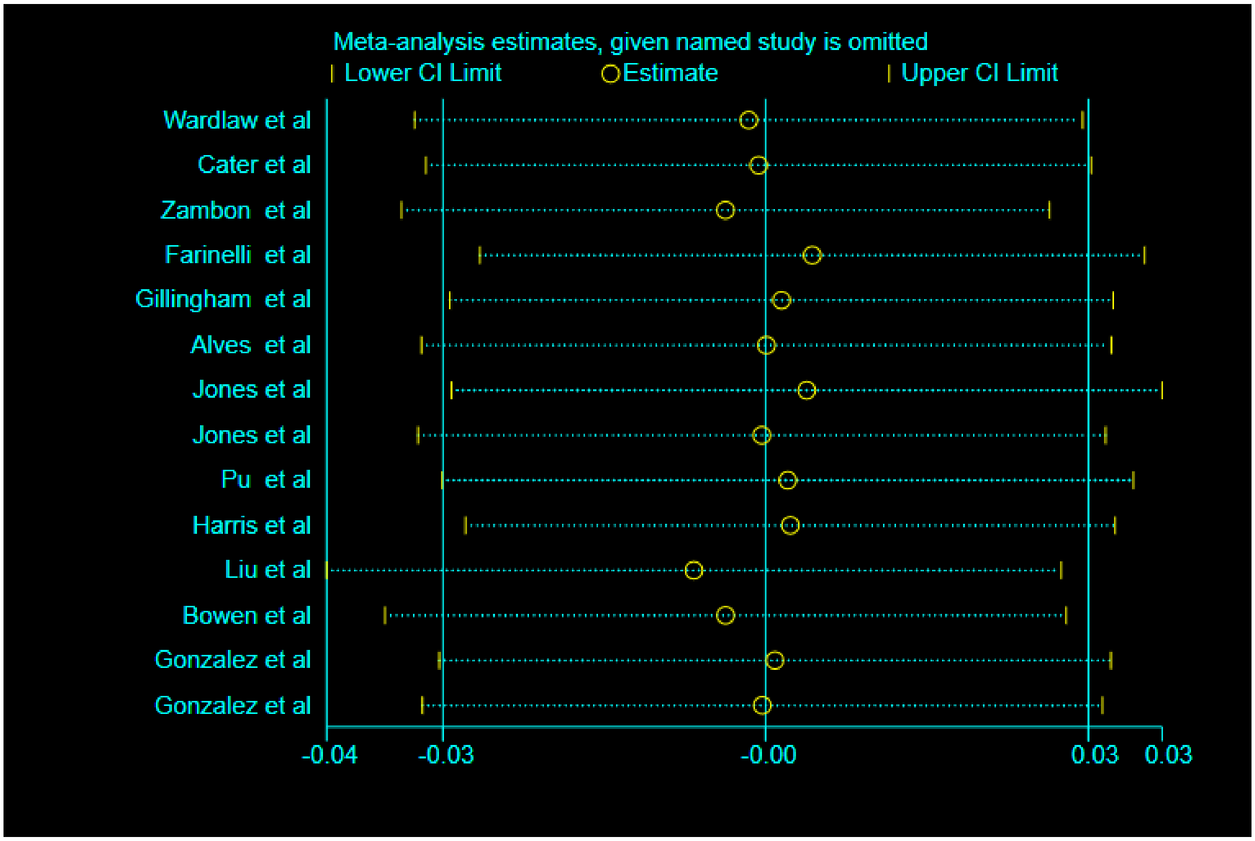

Supplementary Figure 4. TG

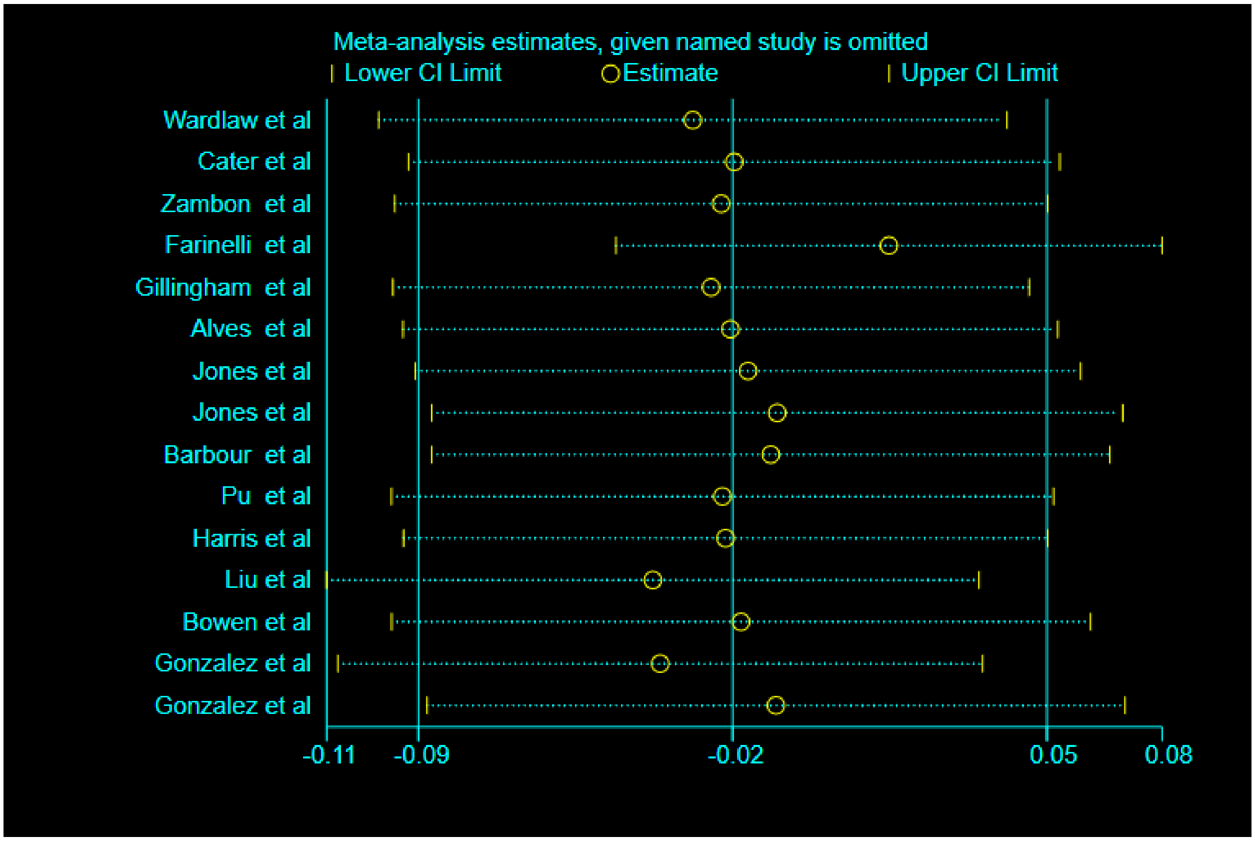

Supplementary Figure 5. FBS

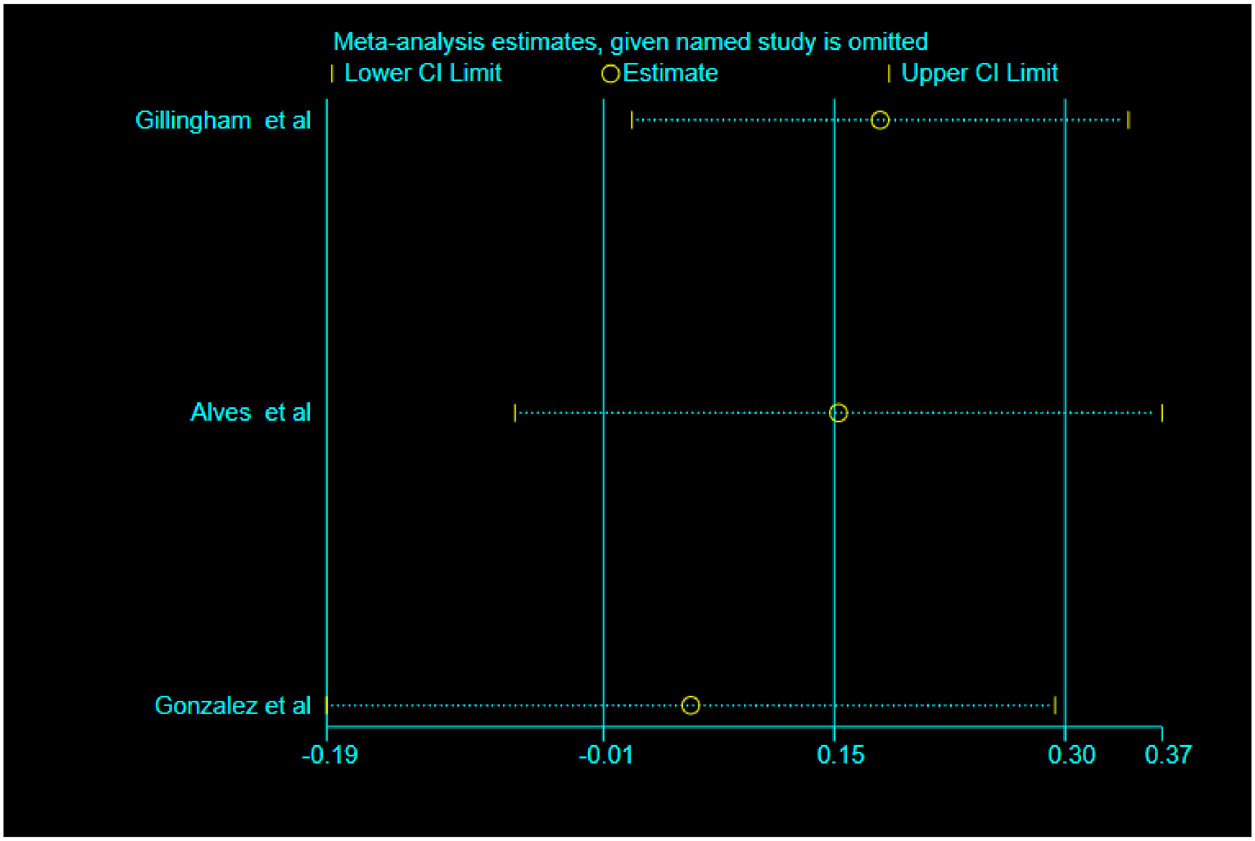

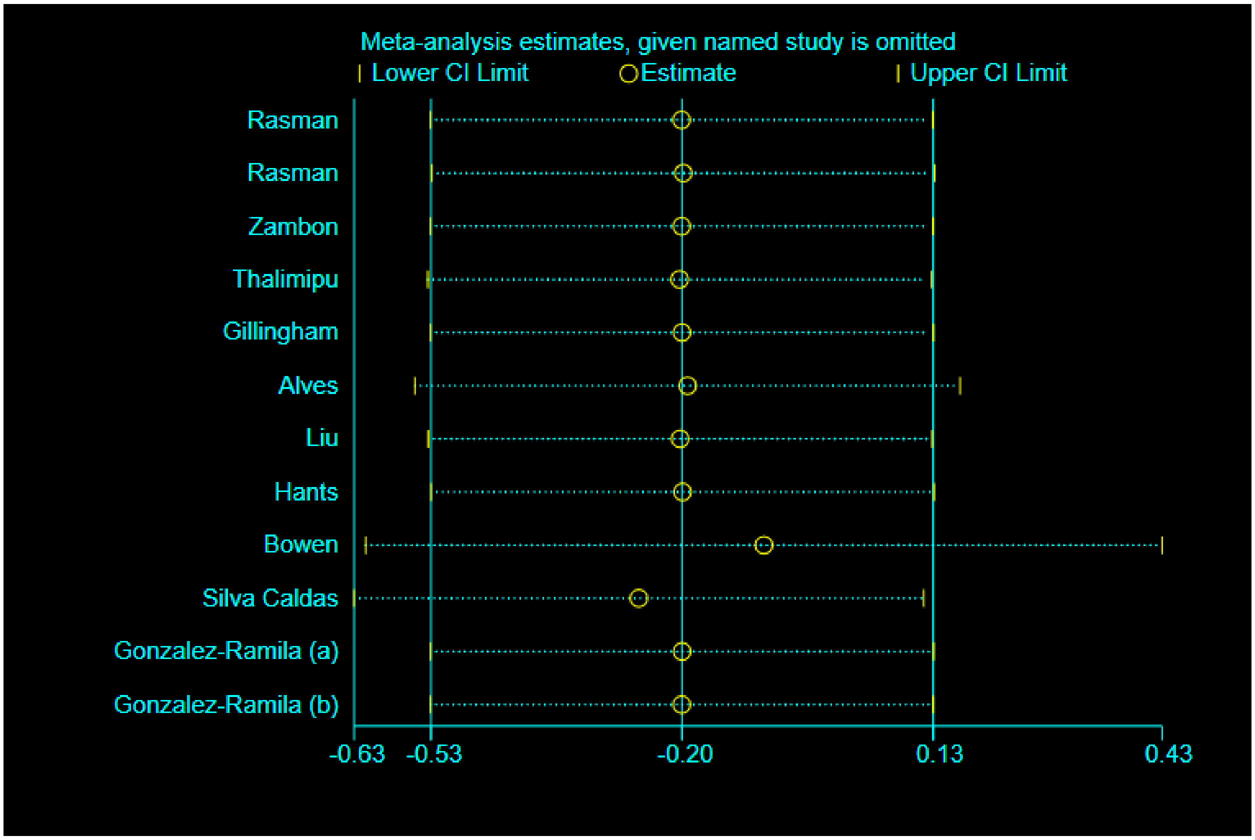

Supplementary Figure 6. Weight

Supplementary Figure 7. BMI

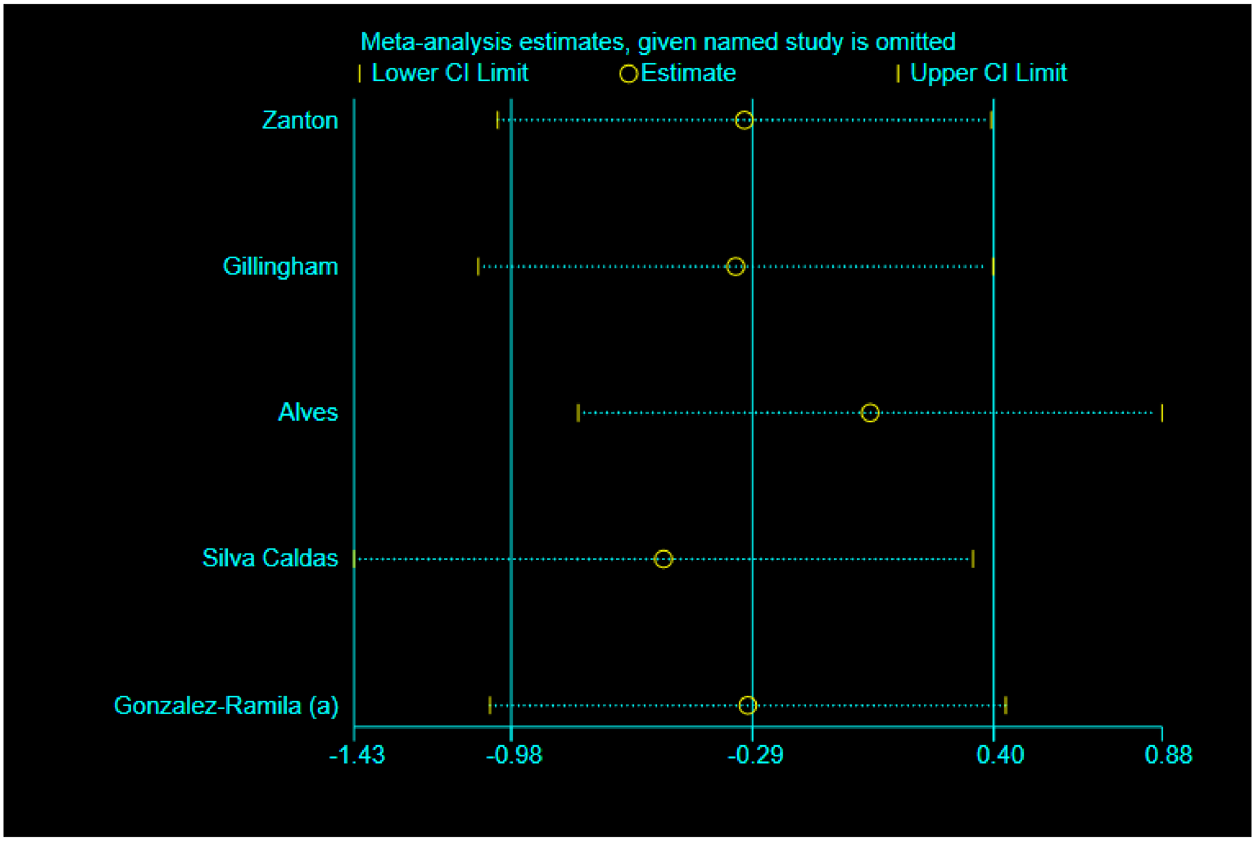

Supplementary Figure 8. SBP

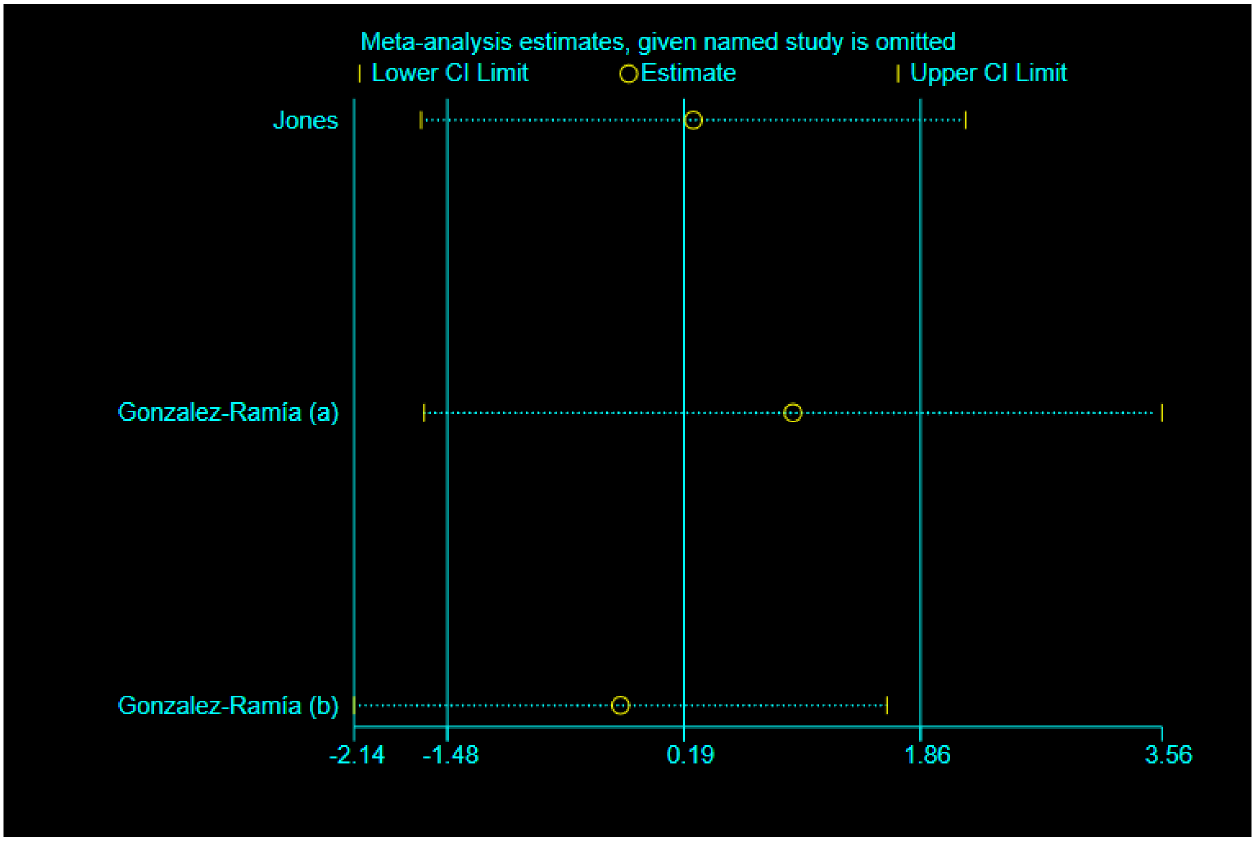

Supplementary Figure 9. DBP

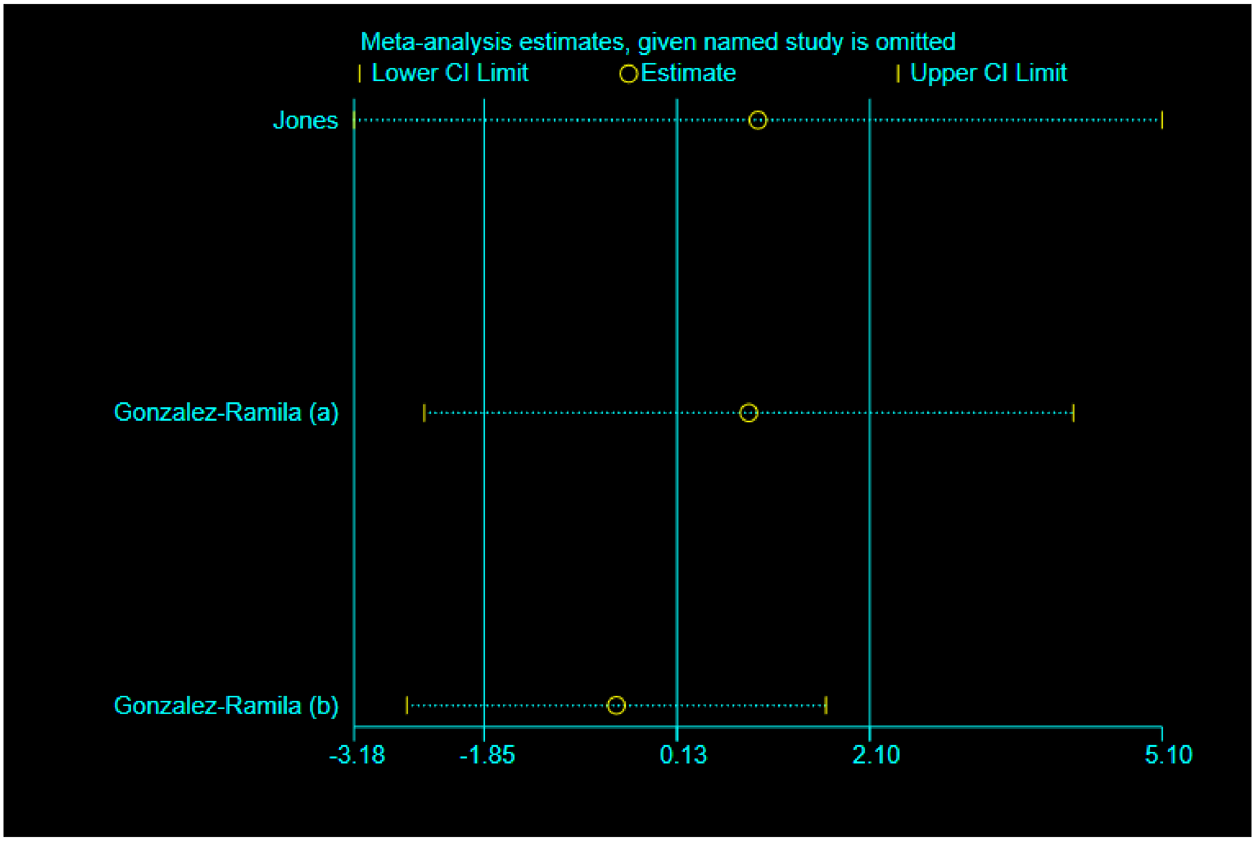

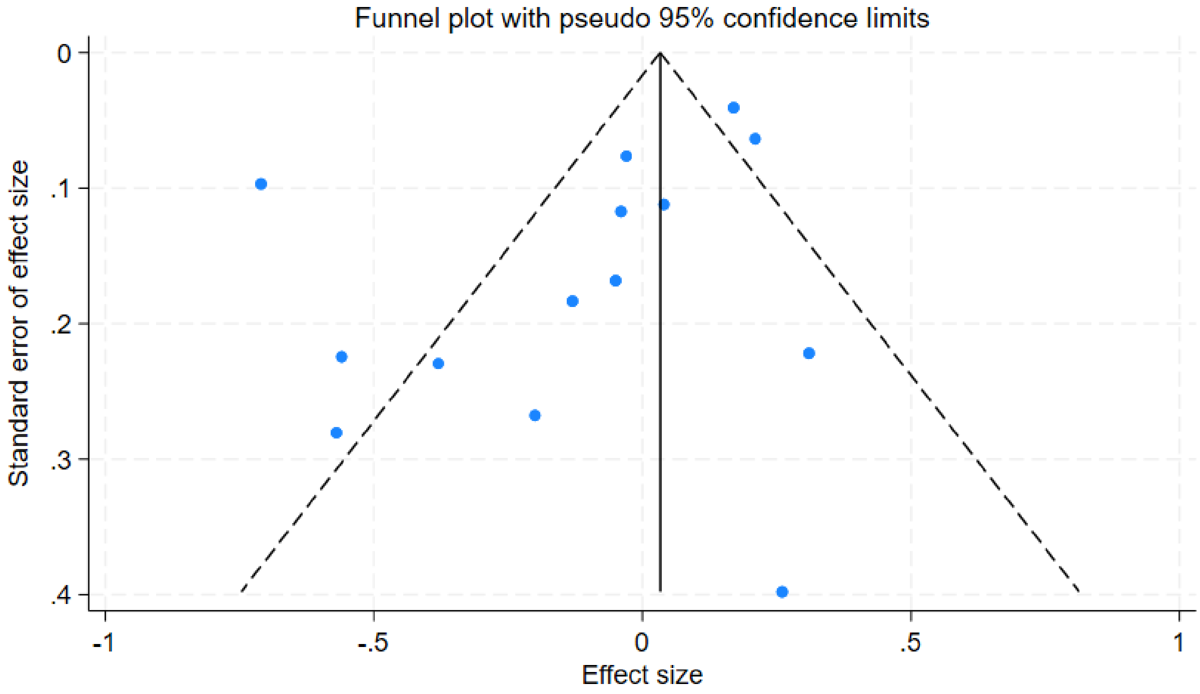

Supplementary Figure 10A. TC

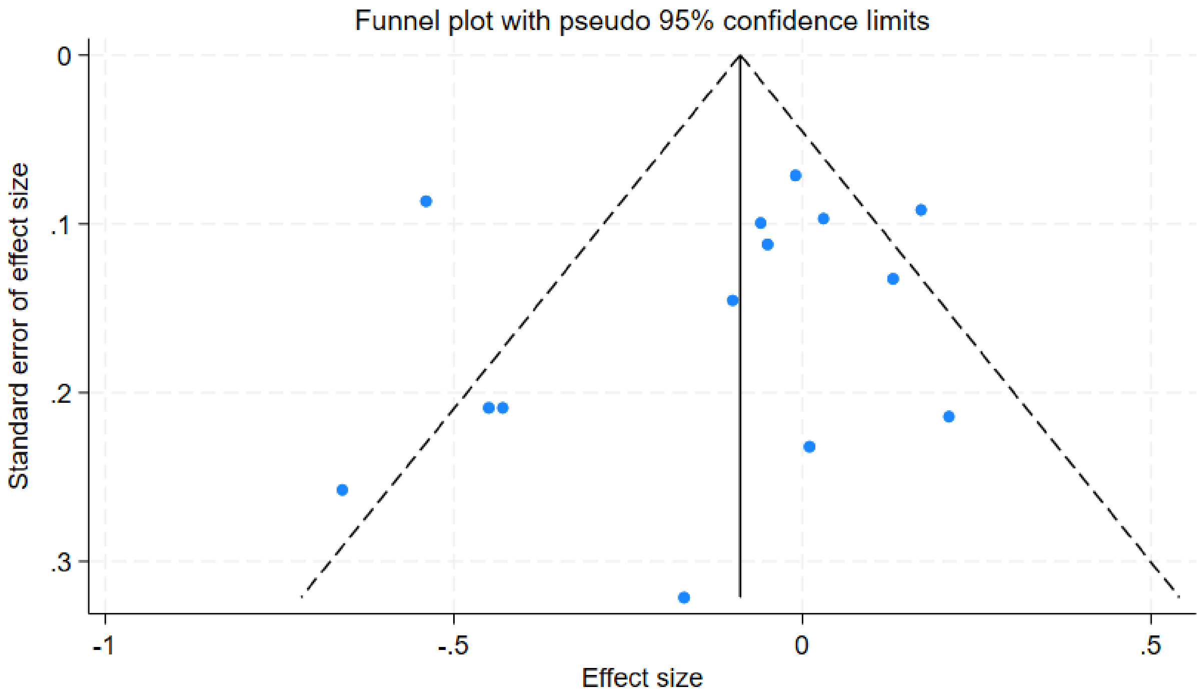

Supplementary Figure 10B. LDL-C

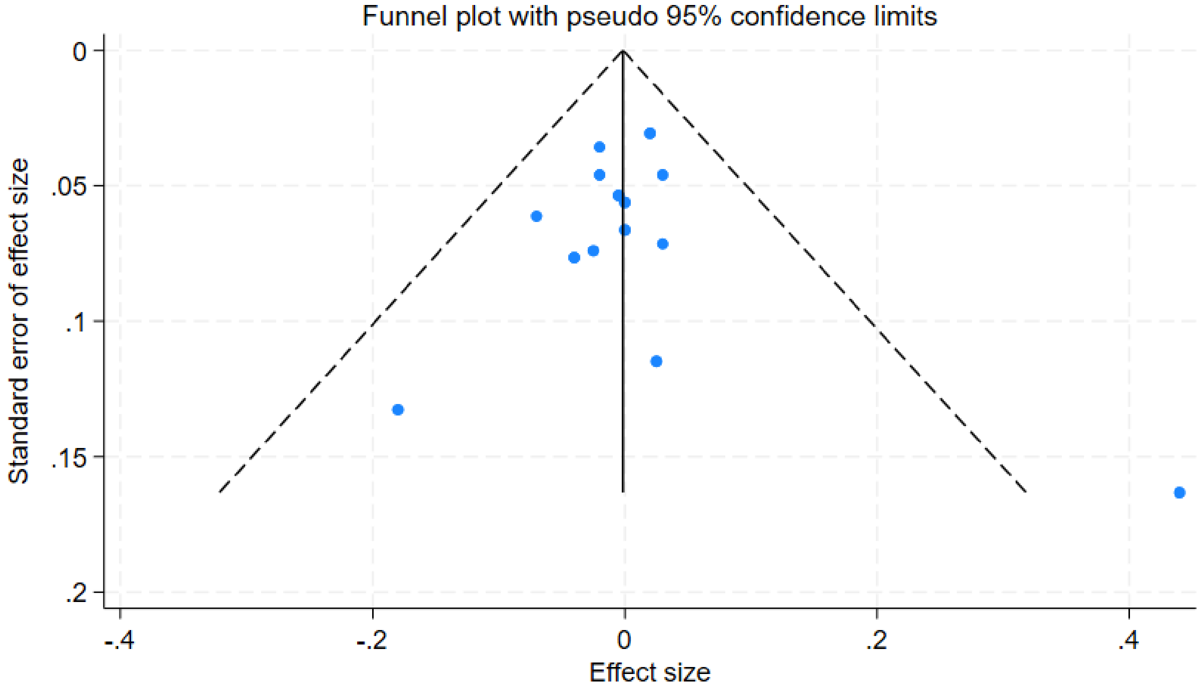

Supplementary Figure 10C. HDL-C

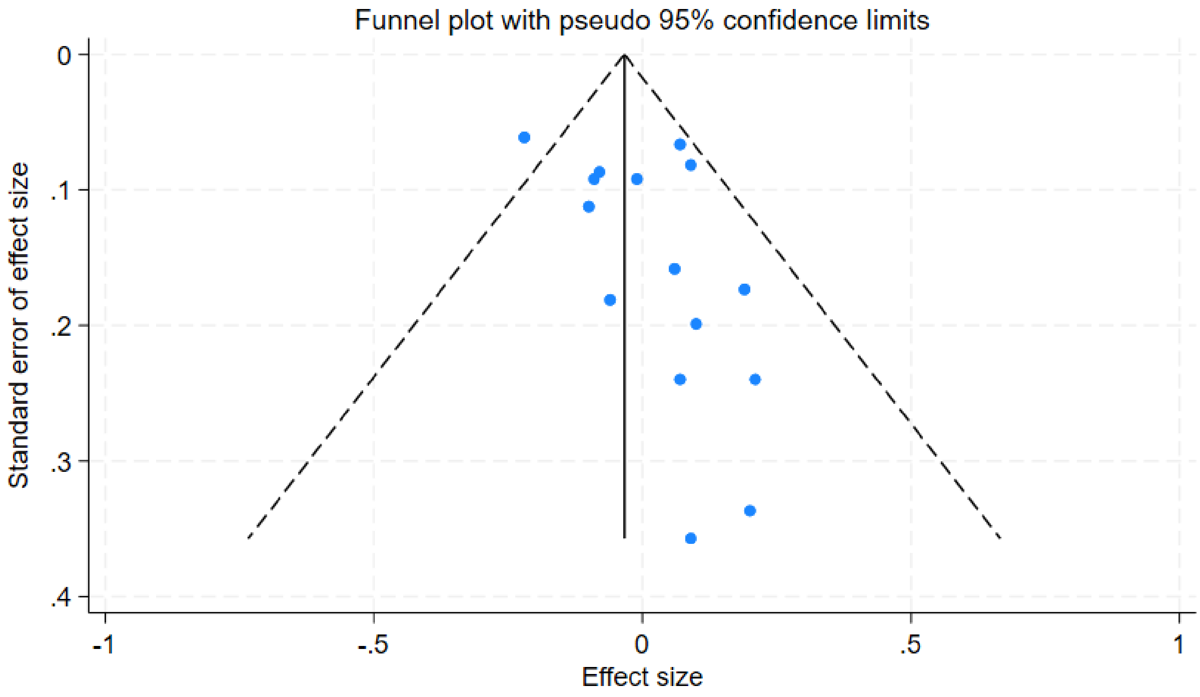

Supplementary Figure 10D. TG

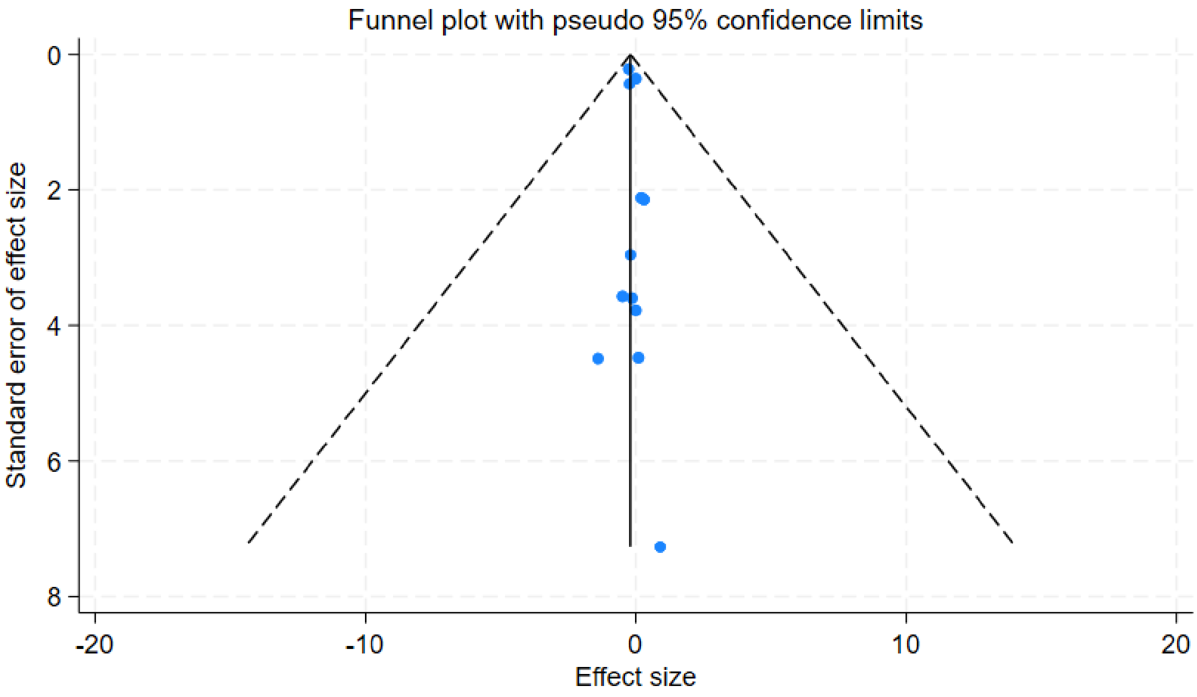

Supplementary Figure 10E. Weight

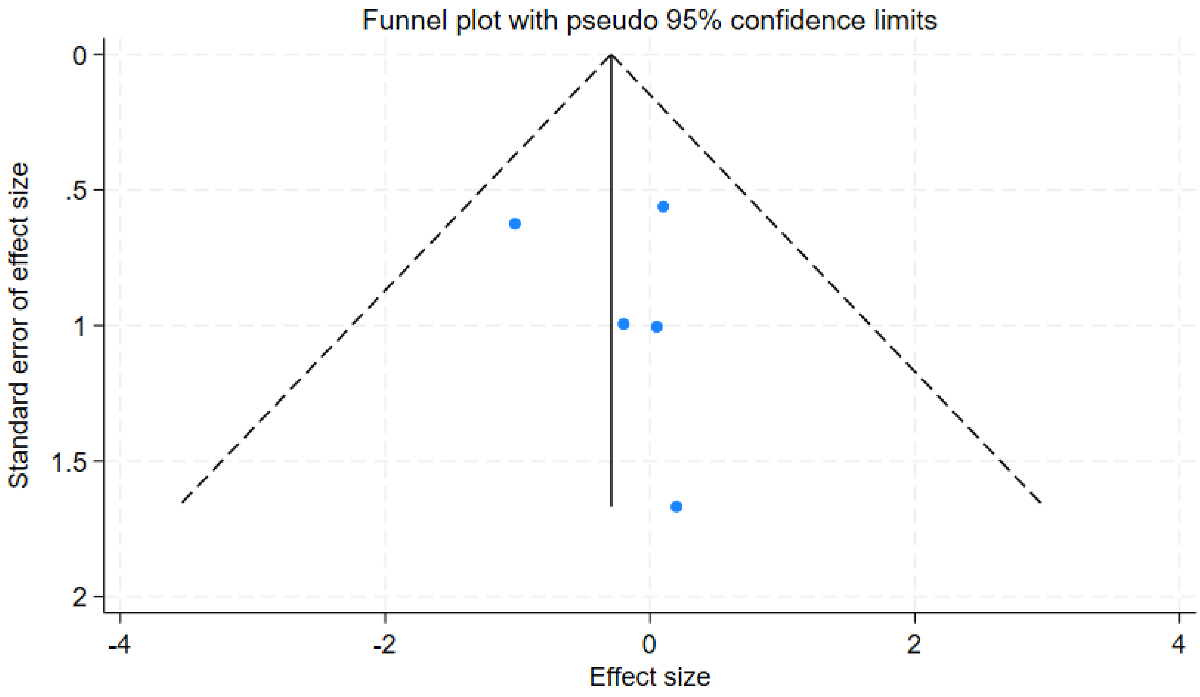

Supplementary Figure 10F. BMI

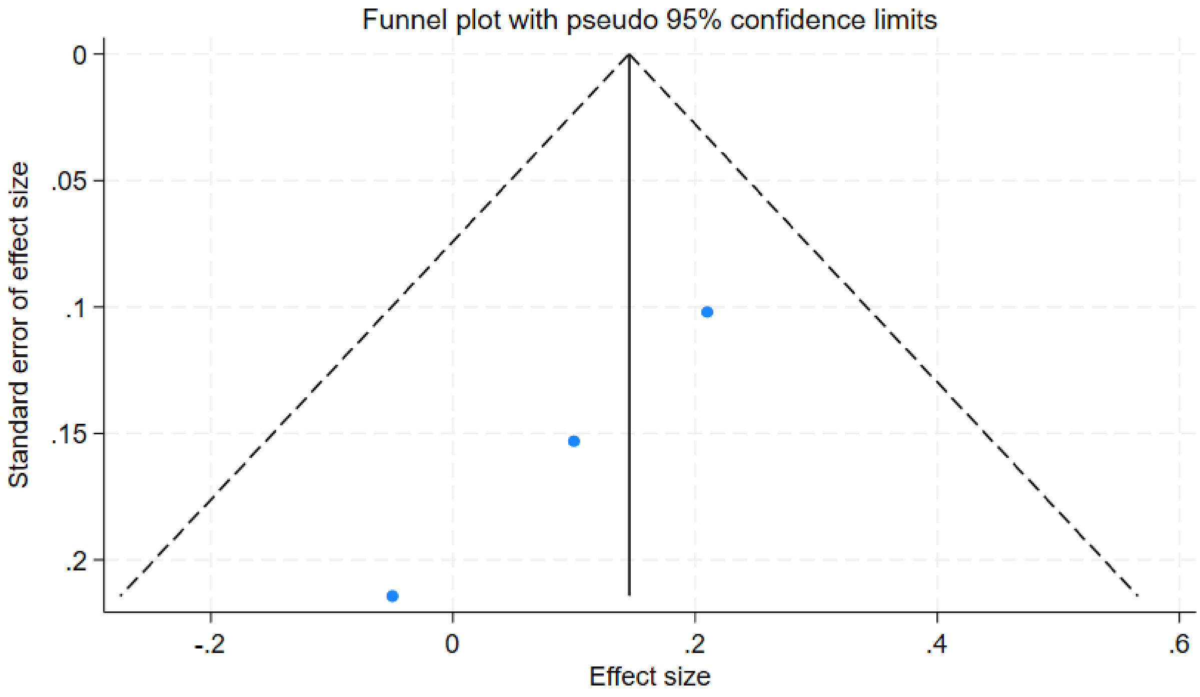

Supplementary Figure 10G. FBS
